# Complications of canine tonsillectomy by clamping technique combined with monopolar electrosurgery – a retrospective study of 39 cases

**DOI:** 10.1186/s12917-022-03342-0

**Published:** 2022-06-24

**Authors:** Outi Marita Turkki, Caroline Elisabeth Bergman, Marcel H. Lee, Odd Viking Höglund

**Affiliations:** 1 AniCura Small Animal Hospital Bagarmossen, Ljusnevägen 17, SE 128 48 Bagarmossen, Sweden; 2 Department of Clinical Sciences, Swedish University of Agricultural Sciences, Box 7054, 750 07 Uppsala, Sweden; 3 Evidensia Södra Djursjukhuset Kungens Kurva, Månskärsvägen 13, Kungens Kurva, 141 75 Huddinge, Sweden

**Keywords:** Canine tonsillectomy, Dog tonsillectomy, Tonsillectomy complication, Tonsillar bleeding, Tonsillar haemorrhage, Tonsillectomy technique

## Abstract

**Background:**

Canine tonsillectomy is performed due to acute or chronic tonsillitis, neoplasia, trauma or occasionally brachycephalic obstructive airway syndrome. Several tonsillectomy techniques are used but information about surgical complications is scarce. This retrospective study of patient records at the University Animal Hospital aimed to investigate complications related to canine tonsillectomy performed by 20-min clamping combined with monopolar electrosurgery.

Inclusion criteria were bilateral tonsillectomy performed with “20-min clamping technique combined with monopolar electrosurgery without suture or ligation”. Exclusion criteria were unilateral tonsillectomy, tonsillar neoplasia, additional surgical procedures other than tonsillectomy, cases where sutures were used initially, and cases where unspecified or other methods of tonsillectomy were used. The search of the patient records of the University Animal Hospital included a 10-year period. Complications that required additional anaesthesia were defined as major complications. Minor complications were handled during surgery or after surgery without surgical intervention.

**Results:**

Of 39 dogs that fulfilled the inclusion criteria, 11 dogs had complications and out of those 1 dog had two complications. Altogether, of the 12 complications, 2 were classified as major complications and 10 as minor.

The most frequent complication was bleeding from the surgical site, in total 11 incidences; 10 dogs had an incidence of bleeding and out of those, 1 dog bled twice, both during and after surgery. Of these 10 dogs that bled, seven incidences of bleeding occurred during surgery and four incidences occurred after surgery. The two dogs with major complications were re-anaesthetized due to bleeding after surgery. No lethal complications occurred and all dogs survived to discharge.

**Conclusions:**

Bleeding during and after surgery was a common complication in dogs after bilateral tonsillectomy using “20-min clamping technique combined with monopolar electrocautery”. Revision intervention was often needed, sometimes urgently. Although no comparison was made with another technique, the studied technique should be used with caution.

## Background

Tonsillectomy in dogs is indicated for management of acute or chronic tonsillitis, trauma, neoplasia and occasionally with brachycephalic obstructive airway syndrome [1, 2]. Different surgical techniques are described in veterinary literature [1, 3, 4]. Usually, a short-time clamping of the tonsils’ adjacent tissue with haemostat combined with suture or manual ligation or electrosurgical tissue sealing are most commonly used techniques. The complication rate after tonsillectomy in dogs is suggested to be low and evidence is scarce [1, 3, 4]. The technique of forceps clamped across the tonsillar base for 20 min is widely used in Sweden. However, haemostasis primarily relies on crushed tissue, and its efficacy and related complications are not reported.

The studied surgical technique, here called “20-min clamping technique combined with monopolar electrosurgery”, is a modified standard technique without suture where tonsillar tissue is grasped with forceps and clamps are placed across the tonsillar base for 20 min before transection of tissue. The transection is done with monopolar diathermy or scissors combined with monopolar diathermy.

A study by Belch et al. [5] evaluated the effectiveness of a vessel-sealing device with bipolar technique, the short 5-mm LigaSure device (Covidien Inc., USA) and compared it to a standard technique with tonsillectomy forceps (Veterinary Instrumentation, Sheffield, UK) combined with the Parker-Kerr suture in 20 dogs suffering from brachycephalic obstructive airway syndrome (BOAS). The techniques were compared on the same dog, one technique on each side of the larynx [5]. When the LigaSure device was used (*n* = 20 tonsils) no additional sutures were needed for haemostasis. However, in one dog the LigaSure device was reapplied to control bleeding. With the standard Parker-Kerr technique, in 8 of 20 tonsils an additional suture was needed due to bleeding. Additionally, in three dogs the tonsillectomy clamp slipped after transection, and in one dog the clamp technique was abandoned due to limited space and therefore the crypt was sutured without clamp. Belch et al. reported no postoperative bleeding [5].

Cook et al. [6] used the bipolar Small Jaw LigaSure Instrument (Covidien Inc., USA) and all 22 BOAS related tonsillectomies were completed without bleeding complications during or after surgery. In a study by Eesa [7] which included 11 dogs, a traditional ligation technique was compared to an electrocautery technique with or without use of an endoscope. One minor bleeding during surgery was reported when traditional ligation was used [7].

The objective of this retrospective study was to investigate the incidence of complications during and after surgery in canine tonsillectomy performed with 20-min clamping technique combined with monopolar electrosurgery without sutures by using the patient records at the University Animal Hospital, Swedish University of Agricultural Sciences, Uppsala (SLU). We expected that the complication rate would be low and that the most common complication would be bleeding.

## Results

Sixty-six cases of canine tonsillectomies were identified in the database, but only 39 dogs met the inclusion criteria.

There were 23 males and 16 females, of which 3 males and 1 female were gonadectomized. The median age of dogs was 2.9 years (IQR 1.6 – 5.7) at time of surgery. The three most common breeds were Standard Poodle (*n* = 4), Irish Setter (*n* = 3), Dutch hound (*n* = 3), three of the dogs were mixed breeds. Median body weight was 18.2 kg (IQR 9.0 – 28.2): 14 dogs weighed less than 10 kg, 17 dogs weighed 10 to 30 kg and 8 dogs weighed more than 30 kg. The main indication for tonsillectomies was chronic tonsillitis (*n* = 36), followed by acute tonsillitis (*n* = 2) and acute trauma with ongoing history of chronic tonsillitis (*n* = 1). Median clamping time was 21 min (IQR 20 – 30).

### Complications

In total, 12 incidences of complications were found; 11 of bleeding and 1 of coughing with signs of laryngeal pain after surgery. One dog had bleeding both during and after surgery. Of these 12 incidences, seven were during surgery and five after surgery (Table 1).

**Table 1:** Minor and major surgical complications after 39 canine tonsillectomies

Complication	Total	Minor	Major
**Total**	12	10	2
Intraoperative bleeding	7	7^a^	-
Postoperative bleeding – no surgery treatment	2	2^a^	
Postoperative bleeding – surgery required	2	-	2
Total incidences of bleeding	11		
Signs of pharyngeal pain and coughing at day four	1	1	-

^a^One dog belonged to both groups; bleeding during and after surgery without revision surgery

### Minor complications

Ten (26%) minor complications were reported in 9 out of 39 dogs, of which seven occurred during surgery and three after surgery. Bleeding during surgery was controlled by suturing the tonsillar fold (*n* = 4) over the empty crypt with a simple interrupted or continuous suture pattern or a combination of manual ligation and tonsillar fold suturing (*n* = 2) or the use of monopolar diathermy (*n* = 1). Poliglecaprone 25 (Monocryl, Ethicon) was used as a ligation or suture material in all cases.

Bleeding after surgery occurred during hospitalisation in two dogs. They were treated without surgical intervention. In both cases with oral bleeding, the bleeding stopped and haemostasis was verified. In one of these dogs bleeding was observed 10 min after recovery from anaesthesia and diminished within one hour without surgical intervention. The dog was treated with a single dose of 10 mg/kg iv. tranexamic acid (Cyklokapron®, Pfizer). The other dog showed bloody salivation directly after anaesthesia, but signs of oral bleeding disappeared after a few hours without any treatment. A third dog with minor complications after surgery was coughing with signs of laryngeal pain on the fourth day after surgery. The dog was presented on a recheck and treated with non-steroid anti-inflammatory medication 2 mg/kg twice a day po. (Rimadyl® vet., Zoetis) for five days. Laryngeal signs resolved.

### Major complications

Two (5%) major complications occurred in two out of 39 dogs with bleeding from the tonsillar vessels. In one of them, haemostasis was achieved by a combination of clamping for 20 min and monopolar diathermy. In the other dog that was re-anesthetized, the tonsillar fold was sutured and the vessel was ligated. Both dogs recovered uneventfully.

## Discussion

Complications after canine tonsillectomy without suturing were investigated, and the most common complication during and after surgery was bleeding from the surgical site. Although no fatal complications were found in the study population, the identification of 10 (26%) minor and 2 (5%) major complications, with a total of 11 incidences of bleeding out of 39 cases raises concerns about the use of the technique.

This study found a higher incidence of bleeding, 11 incidences in 39 dogs, compared to previous studies using energy-based vessel sealing devices [5, 6]. In the study by Belch et al. the LigaSure device had to be reapplied once in 20 tonsils. Cook et al. also used the LigaSure device and reported no bleeding in 22 dogs. The present technique, with over 20 min of clamping of the tonsillar base, indicated a longer duration of surgery compared to Belch’s use of LigaSure with less than one minute for seal and cut [5]. The present study involved electrosurgery but use of the technique was not standardised, which is different compared to use of energy based vessel sealing devices such as LigaSure. Considering the results of the present study and the available data from other studies, a preference for the use of standardised energy-based techniques, vessel sealing devices, may be suggested for tonsillectomy. However, a randomized prospective study to evaluate advantages and drawbacks of each technique should be done.

In this study material the main indication for tonsillectomy was clinically suspected tonsillitis, mainly chronic. It may be that patients with inflamed tonsillar tissue might bleed more easily compared to dogs were laryngeal obstruction is the main reason for surgery. Palatine tonsil is divided into a caudal protruding portion and deeper rostral minor portion. The deep portion is usually absent on young dogs but may be formed later as a result of tonsillitis. The tonsillar artery enters to the middle or widest part of the tonsil and is divided to approximately three branches [8].

Tonsillectomy is a common surgical procedure in humans. Considering species differences, comparison between human and canine studies should be done cautiously. Furthermore, surgical facilities and perioperative care may also differ substantially. Large retrospective studies of human data reported a wide range of complications such as bleeding from the surgical site, superficial and deep surgical site infection, wound rupture, upper respiratory obstruction, dehydration, postoperative pain and inadequate oral nutrition as commonly recognized complications [9–11]. A study by Seshamani et al. [12] reported postoperative complication rate for human tonsillectomies to be 20%, mainly haemorrhage, dehydration and pain, whereas another study by Chen et al. [9] reported a lower rate, 1.2%, mainly infections.

In several reported human case series, the rate of post tonsillectomy bleeding ranged from 2.0% to 7.0% [10, 12–19]. However, the definitions of complications varied slightly. Sarny et al. reported higher rates of post tonsillectomy bleeding, from 14 to 16% in four different studies [20–23]. There is conflicting evidence about surgical technique in studies evaluating the risk for bleeding after tonsillectomy. A meta-analysis by Francis et al. [16] reported that frequency of bleeding after tonsillectomy across different techniques did not differ. Krishna et al. [24] reported benefits for monopolar surgery but two other studies described higher risk of bleeding for “hot” tonsillectomy techniques, when diathermy was used for dissection, compared to traditional “cold steel” [19, 25].

The present study had several limitations. Surgeries were performed by different surgical teams and reports of complications were therefore inconsistent. The decision to treat bleeding during and after surgery was at the discretion of the surgeon and may have differed between surgeons, which influenced the classification of a complication. Furthermore, an incident may not be regarded as a complication, but rather an expected sequela of surgery. Other limitations were the small number of dogs, weight variation, age and type of dogs. An additional limitation was the lack of long-term follow-up; owners of the dogs were not contacted and potential late complications may have been treated at another clinic.

This study was not designed to evaluate or compare safety of different techniques. Due to the involvement of several different surgeons and the retrospective nature of the study, the results of the present study should be used cautiously in recommendation of any particular surgical technique. A prospective randomized study comparing several techniques is needed.

## Conclusions

Bleeding during and after surgery was a common complication in dogs after bilateral tonsillectomy using “20-min clamping technique combined with monopolar electrocautery”. Revision intervention was often needed, sometimes urgently. Although no comparison was made with another technique, the studied technique should be used with caution.

## Methods

### Data collection

The patient record database at SLU was screened for dogs that had tonsillectomy performed from Jan 1st, 2007 to Dec 31st, 2016. Retrieval of patient data was done six months after the most recent surgery.

Inclusion criteria was bilateral tonsillectomy performed by using 20-min clamping combined with monopolar electrosurgery, without suture or ligation. Exclusion criteria were unilateral tonsillectomy, tonsillar neoplasia, additional surgical procedure other than tonsillectomy, cases where sutures were used initially, and cases where unspecified or other methods of tonsillectomy were used.

After search through patient records, the following data was collected: breed, age, body weight, sex and neuter status, indication for surgery and complications during and after surgery up to one week [26]. Furthermore, duration of clamping time with haemostat, possible treatment interventions due to complications during and after surgery were also recorded. Body weight, age and clamping time were presented as median and interquartile range (IQR).

### Complications

Minor complications were defined as complication that were handled during or after surgery and did not require a new surgical intervention. Major complications were defined as complications that required re-anaesthetizing the patient for revision surgery. Major and minor complications were reported as cumulative incidence (%).

## Data Availability

Corresponding author can be requested for detailed data used for the study.

## Abbreviations

BOAS: Brachycephalic obstructive airway syndrome
n: Number
IQR: Interquartile range
iv.: Intra venous
po.: Per oral
